# How Different Albumin-Binders Drive Probe Distribution of Fluorescent RGD Mimetics

**DOI:** 10.3389/fchem.2021.689850

**Published:** 2021-08-24

**Authors:** Carsten Höltke, Wael Alsibai, Martin Grewer, Miriam Stölting, Christiane Geyer, Michel Eisenblätter, Moritz Wildgruber, Anne Helfen

**Affiliations:** ^1^ Clinic for Radiology, University Hospital Münster, Münster, Germany; ^2^ Department of Radiology, University Hospital Freiburg, Freiburg, Germany; ^3^ Department of Radiology, University Hospital, LMU Munich, Munich, Germany

**Keywords:** RGD-mimetics, integrin imaging, optical imaging, albumin, bioavailability, tumor imaging

## Abstract

The biodistribution of medical imaging probes depends on the chemical nature of the probe and the preferred metabolization and excretion routes. Especially targeted probes, which have to reach a certain (sub)cellular destination, have to be guided to the tissue of interest. Therefore, small molecular probes need to exhibit a well-balanced polarity and lipophilicity to maintain an advantageous bioavailability. Labelled antibodies circulate for several days due to their size. To alter the biodistribution behavior of probes, different strategies have been pursued, including utilizing serum albumin as an inherent transport mechanism for small molecules. We describe here the modification of an existing fluorescent RGD mimetic probe targeted to integrin α_v_β_3_ with three different albumin binding moieties (ABMs): a diphenylcyclohexyl (DPCH) group, a *p*-iodophenyl butyric acid (IPBA) and a fatty acid (FA) group with the purpose to identify an optimal ABM for molecular imaging applications. All three modifications result in transient albumin binding and a preservation of the target binding capability. Spectrophotometric measurements applying variable amounts of bovine serum albumin (BSA) reveal considerable differences between the compounds concerning their absorption and emission characteristics and hence their BSA binding mode. *In vivo* the modified probes were investigated in a murine U87MG glioblastoma xenograft model over the course of 1 wk by fluorescence reflectance imaging (FRI) and fluorescence mediated tomography (FMT). While the unmodified probe was excreted rapidly, the albumin-binding probes were accumulating in tumor tissue for at least 5 days. Considerable differences between the three probes in biodistribution and excretion characteristics were proved, with the DPCH-modified probe showing the highest overall signal intensities, while the FA-modified probe exhibits a low but more specific fluorescent signal. In conclusion, the modification of small molecular RGD mimetics with ABMs can precisely fine-tune probe distribution and offers potential for future clinical applications.

## Introduction

Integrins are heterodimeric cell surface proteins consisting of different α and β subunits with various contributions to cell–cell and cell–extracellular matrix (ECM) interactions (Hynes, 2002). They are described to regulate cell structure and behavior by affecting cell differentiation, migration and survival (Moreno-Layseca et al., 2019). As a result, a dysregulated expression is associated with a variety of diseases, including cancer (Hamidi and Ivaska, 2018). In particular, α_v_β_3_ plays an important role in the regulation of normal and tumor cell proliferation and survival, in tumor angiogenesis and in the metastatic potential of tumors (Desgrosellier and Cheresh, 2010; Weis and Cheresh, 2011). Therefore, α_v_β_3_ is an important target in noninvasive tumor detection and for monitoring therapy (Su et al., 2020). A high affinity to the Arg-Gly-Asp (RGD) peptide motif, which is found in ligands like fibronectin, vitronectin or fibrinogen is a common feature of many integrins (Hynes et al., 2002; Humphries et al., 2006). Consequently, synthetic peptides containing the RGD motif and peptidomimetic structures based on RGD have been designed and utilized for therapeutic, but also molecular imaging approaches (Marelli et al., 2013; Höltke and Faust, 2017).

We introduced a small molecular RGD-mimetic with high affinity and selectivity for the optical imaging of integrin α_v_β_3_ expression (Alsibai et al., 2014). It was constructed of a 2-amino-tetrahydropyrimidine as Arg-mimetic, a central aromatic group, a diaminopropionic acid as Asp-mimetic, a spacer attached to a peripheral aromatic group and a cyanine dye for detection by fluorescence imaging. The hydrophilicity of the whole construct resulted in a very rapid elimination *in vivo* with half-lives of distribution and excretion of less than 50 min and 10 h, respectively. Hence, the imaging performance of the compound was less favorable. Therefore, we sought for a method to improve bioavailability and imaging performance by modifying the molecular structure of the ligand.

Serum albumin is the most abundant protein in plasma, with a concentration of up to 750 μM. It has a net mass of 66.5 kDa, a high cystein-content and a rather acidic character, which makes it profoundly soluble and highly robust. In plasma it regulates oncotic pressure and pH and binds and transports molecules, drugs and ions, among others (Merlot et al., 2014). Albumins can non-covalently bind a large variety of endogenous and exogenous compounds and thereby enhance the bioavailability of these substances (Leboffe et al., 2020). Especially tumors represent a promising target for albumin-based therapies (Sleep, 2015). The vasculature inside tumors is often characterized by wide fenestrations resulting from undamped angiogenesis with a lack of a functional endothelial layer. In addition, tumors do not grow an effective lymphatic system responsible for draining cell debris and extracellular waste. This leads to an erratic blood flow inside tumor lesions and an enhanced retention of larger molecules. This enhanced permeability and retention (EPR) effect has widely been used in approaches to use larger molecules (serum proteins as well as artificial drug carriers) as vehicles for the delivery of therapeutics and imaging agents (Maeda, 2015; Liu and Chen, 2016). A number of approaches have already been translated to clinical practice. Abraxane^®^, a paclitaxel-albumin nanoparticle, is used as a therapeutic in metastatic breast cancer, advanced pancreatic cancer, and non-small cell lung cancer (Yardley, 2013; Blair and Deeks, 2015). Aldoxorubicin, which is a maleimide and acid-cleavable hydrazone prodrug of doxorubicin, binds covalently to the cysteine-34 position of circulating albumin and is converted in acidic environment like the lysosome or in cancerous milieu. A phase III clinical trial, where this compound was used for the treatment of soft tissue sarcoma was completed in 2017 (Kratz, 2008; Chamberlain et al., 2019). Strategies to utilize albumin binding for medical imaging already include compounds for nuclear imaging and magnetic resonance imaging in clinical routine. Technetium ^99m^Tc macro aggregated albumin (^99m^Tc-MAA) is an injectable radiopharmaceutical agent used for lung perfusion imaging and hepatic arteriography before radioembolization with ^90^Y-microspheres to identify gastrointestinal shunts (Garin et al., 2019). Vasovist^®^ (MS-325, Gadofosveset) is a blood-pool contrast agent used in magnetic resonance angiography for the detection of aortoiliac occlusive disease by transient non-covalent binding to albumin (Parmelee et al., 1997). In more preclinical settings a number of radionuclide-labeled tracers have been developed and tested in rodent models of human cancers. These often include a chelating moiety for radiometals to enable imaging and therapy with the same molecule (theranostic) and represent a class of dual targeting constructs with one “target” being the tumor EPR-effect and the other being a tumor therapeutic effector like PSMA (Bouchelouche et al., 2016; Rahbar et al., 2016; Jadvar et al., 2018; Deberle et al., 2020).

In this work, we identified the effects of different albumin binding moieties (ABMs) on the biodistribution and tumor targeting capability of small molecular fluorescent RGD-mimetics with the goal to enhance the bioavailability of the probes at the target site by retardation of metabolism and excretion. We synthesized three probes with different ABMs assembled on the same integrin α_v_β_3_-targeted small molecule with the hypothesis, that one of the modified probes should at best show superior targeting and imaging performance, defined by a high target-to-background ratio. A fatty acid (FA) like in Levemir^®^ (Keating, 2012), a diphenylcyclohexyl-group (DPCH) as used in Vasovist^®^ (Bremerich et al., 2007) and a *p*-iodophenylbutyric acid (IPBA) moiety as introduced by Dumelin et al. (Dumelin et al., 2008) were attached for this purpose. We compared the findings to examinations with the original, unmodified probe, which comprises the same targeting structure and the same fluorescent dye but no ABM.

## Materials and Methods

*General*. All chemicals, reagents and solvents for synthesis were analytical grade and purchased from commercial sources. Bovine serum albumin (BSA) was from Sigma Aldrich (St. Louis, MI, United States). The syntheses of the amino-polyethylene glycol (PEG) precursors **1** and **1'** and of the unmodified probe **2** are described in previous work (Alsibai et al., 2014; Hahnenkamp et al., 2014). The detailed syntheses of the DPCH-modified probe **3**, the fatty acid (FA)-derived probe **4** and the *p*-iodophenylbutyric acid (IPBA)-modified probe **5** are described in the supporting information section. Mass spectrometry was performed using a Waters QUATTRO LCZ (Waters Micromass, Manchester, United Kingdom) or an Orbitrap LTQ XL (Thermo Scientific, Dreieich, Germany) spectrometer with nanospray capillary inlets. HPLC-purification was performed on a gradient RP-HPLC using a Shimadzu Prominence system (Shimadzu Deutschland GmbH, Duisburg, Germany), acetonitrile and purified water containing 0.1% trifluoro acetic acid (TFA) as mobile phases and C18 RP columns (detailed information can be found in the supporting information section).

*Gel electrophoresis*. Agarose gels (1.5% in TAE buffer) were used to determine albumin binding of the different fluorescent probes. An amount of 0.5 nmol of the probes were diluted with PBS containing 1.0% BSA and sample buffer (Fermentas #R0631; Thermo Scientific, Dreieich, Germany) to a total volume of 17.5 µL. Gels were run with 110–130 V for approximately 60 min in running buffer (25 mM Tris, 200 mM glycine) and visualized by fluorescence reflectance imaging (In-Vivo FX Pro Imaging System; Bruker BioSpin GmbH, Rheinstetten, Germany) and subsequent Coomassie blue staining. Images were analysed by Bruker MI SE software (version 7.5.2).

*Photo- and Fluorometer Measurements*. The influence of BSA concentration on the absorption and emission spectra was determined by photometric and fluorometric measurements using a U-3010 spectrophotometer and a F-4500 spectrofluorometer (Hitachi High Technologies Europe GmbH, Mannheim, Germany). The fluorescent probes (equal amounts, 400 or 750 nM) were dissolved in 1 ml of either purified water (MQ) or phosphate buffered saline (PBS) or water or PBS containing different amounts of BSA (0.001–1% w/v or 25 nM–100 µM). For competition experiments, an excess of ibuprofen (Ibu) was added to the solution to final concentrations of 486 µM or 1.86 mM. The absorption and emission spectra (excitation wavelength λex. = 630 nm) were recorded at room temperature with the manufacturers’ software (UV-Solutions/FL-Solutions) and analyzed with GraphPad Prism 7.02 (GraphPad Software, La Jolla, CA, United States).

*Cell binding assays*. A number of 60.000 cells (U87MG glioblastoma, ATCC^®^ HTB-14™) were seeded onto microscopic slides (Superfrost PlusTM adhesion slides; Thermo Scientific, Schwerte, Germany) and incubated overnight at 37°C in 5% CO_2_ humidified atmosphere. Cells were washed with PBS at room temperature and fixated with 4% formaldehyde for 10 min. After washing with PBS cells were incubated with fluorescent probes **2–5** at a concentration of 2.0 µM for 1 h at 4°C in binding buffer (0.5 mM MgCl_2_, 0.5 mM CaCl_2_, 25 µM MnCl_2_ in PBS solution). Cells were then washed twice with cold PBS and stained with DAPI solution (10 min rt). Finally, after another washing step, cells were covered with Fluoromount-G™ (Thermo Scientific) and dried overnight at rt in the dark. For control experiments, simultaneous to the addition of probe, a solution of 200 µM cilengitide (Tocris/Bio-Techne GmbH, Wiesbaden, Germany) in binding buffer was added. Images were captured using an Eclipse 50i microscope (Nikon, Tokio, Japan) and documented by NIS-Elements Br 3.22 software (Nikon).

*Xenografts*. Animal experiments were performed in accordance with the national and European legislation for animal care and experiments and were approved by the animal ethics committee of the Landesamt für Natur, Umwelt und Verbraucherschutz Northrhine Westfalia (regional authority for animal ethics LANUV NRW; License numbers: 84-02.04.2016.A459). Athymic 7–9-wk-old female nude mice (*n* = 42 in total) were obtained from Charles River (Sulzfeld, Germany) and maintained in a pathogen-free animal facility with food and water available ad libitum. Human glioblastoma cell line U87MG was purchased from DSMZ (Braunschweig, Germany) and was cultivated in DMEM glutamax supplemented with 10% FCS. Cells were grown routinely in T75 flasks, incubated at 37°C in a 5% CO_2_ humidified air atmosphere until the cultures were sub-confluent (70–80%); medium was changed every 3–4 days. Before injection the cells were incubated with trypsin/EDTA and resuspended in medium at appropriate concentrations. Mice (9–11-wk-old) were implanted with approximately 3–5 × 10^6^ cells subcutaneously in the right hemithorax through 26-gauge needles and tumours were grown for 10–14 days until they reached a diameter of 4–5 mm.

*In vivo fluorescence imaging*. Near infrared FRI was performed using the In-Vivo FX Pro Imaging System (Bruker BioSpin GmbH, Rheinstetten, Germany) equipped with a 400 W halogen illuminator with Cy 5.5 bandpass excitation (625 ± 18.0 nm) and emission filters (700 ± 17.5 nm). Fluorescence signals were captured with a 4-million-pixel cooled charge-coupled device (CCD) camera equipped with a 10× zoom lens. Mice were anesthetized by isoflurane inhalation and received the Cy 5.5-labeled probe (2.0 nmol/animal; *n* = 4–8) dissolved in saline (150 μl) via the tail vein. Images were captured before and at several time points after probe injection (t = 30 min, 60 min, 3, 6, 24, 48, 72 and 96 h), with an acquisition time of 5 s and identical window settings (binning, f-stop, field of view). For biodistribution studies, animals were sacrificed after 96 h by cervical dislocation. Organs were removed, washed and placed on a Petri dish for fluorescence imaging. Fluorescence images were analyzed and co-registered with the anatomic white light images using the Bruker MI 7.5 software. Subsequent to FRI mice were directly transferred to FMT imaging. FMT studies were performed using the small-animal imaging system FMT 2500 (PerkinElmer, Waltham, MA, United States). Mice were placed inside the imaging chamber under isoflurane anesthesia as described elsewhere (Nowacki et al., 2019). Animal scan times were in the range of 2–5 min, and image reconstruction times were about 1–3 min. Volumes of interest were drawn around the heart region with the highest fluorochrome concentration close to the apex. The whole imaged region was used for comparison of probe concentration.

*Statistics*. Data from *in vivo* imaging including biodistribution analyses were collected from four to eight mice for each probe. Data are displayed as mean ± sem except for tumor-organ ratios (Supplementary Figure S1), where errors are displayed as the maximum mean standard deviation (SD) percentage of the tumor-organ pair. Biodistribution data from FRI were processed within GraphPad Prism and two-way ANOVA analysis, *p* < 0.05 was considered statistically significant.

## Results and Discussion

**FIGURE 1:**
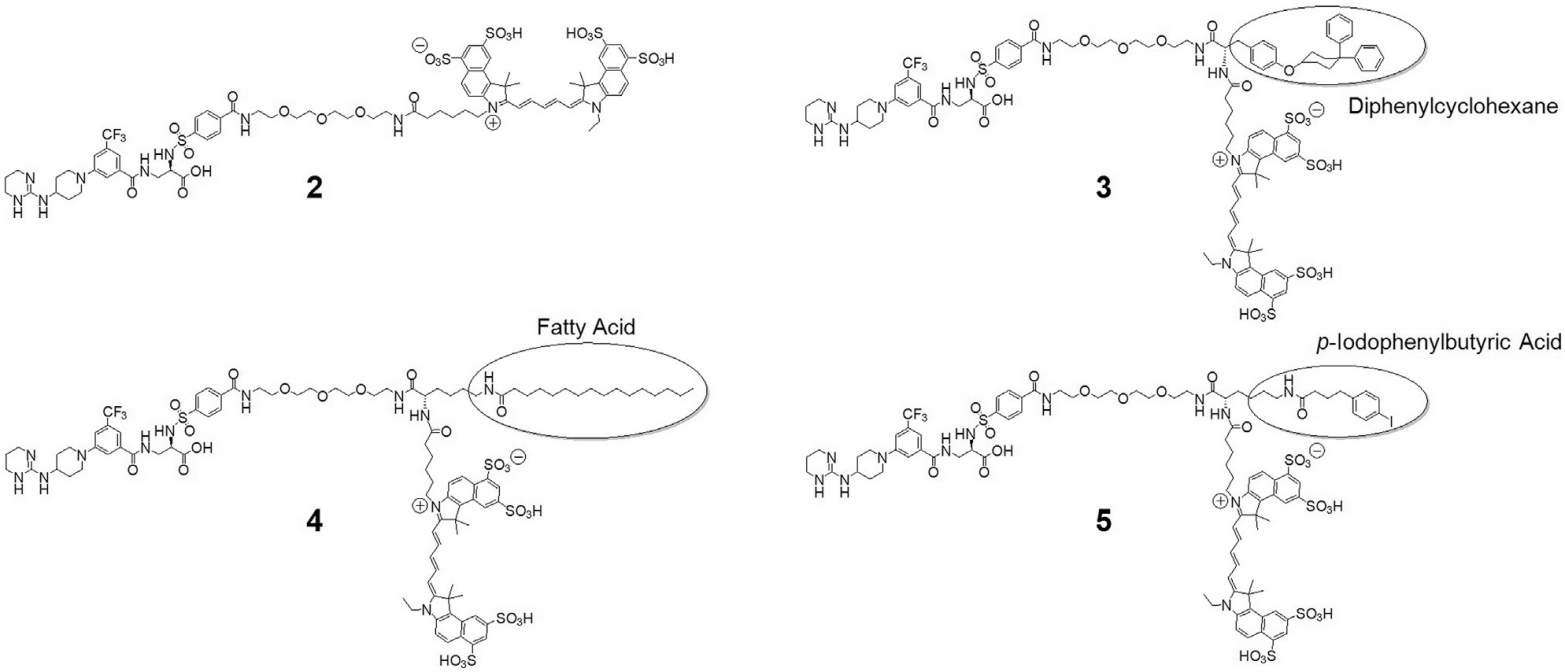
Structures of the developed and investigated probes. Derived from the original probe **2**, we modified the main structure with amino acid linkers (tyrosine and lysine, respectively) to mount albumin binding moieties diphenylcyclohexane to yield **3**, palmitic acid for **4** and *p*-iodophenylbutyric acid for compound **5**. Finally, all probes were labelled with cyanine dye Cy 5.5.

*Chemistry*. The syntheses of the final fluorescent probes were accomplished starting from amino-PEG methyl ester precursor **1** as depicted in the supporting information (Supplementary Scheme S1). The syntheses of the albumin-binding diphenylcyclohexyl and *p*-iodophenylbutyric acid building blocks is described elsewhere (Dumelin et al., 2008; Hahnenkamp et al., 2014). The Boc-protected palmitoyl-lysine derivative is commercially available. After attachment of these building blocks, the methyl ester was saponified (except in the synthesis of **4**, where the free acid was already used) and the Boc-protection was removed by treatment with 4 N HCl in dioxane. These steps were carried out without intermediate purification. Before attachment of the cyanine dye, compounds were purified by HPLC and lyophilized. After dye conjugation HPLC purification of the desired fractions then yielded the final probes, which after lyophilization were reconstituted in pure water for further applications and storage. The structures of the final probes are depicted in Figure 1, modifications are marked.

**FIGURE 2:**
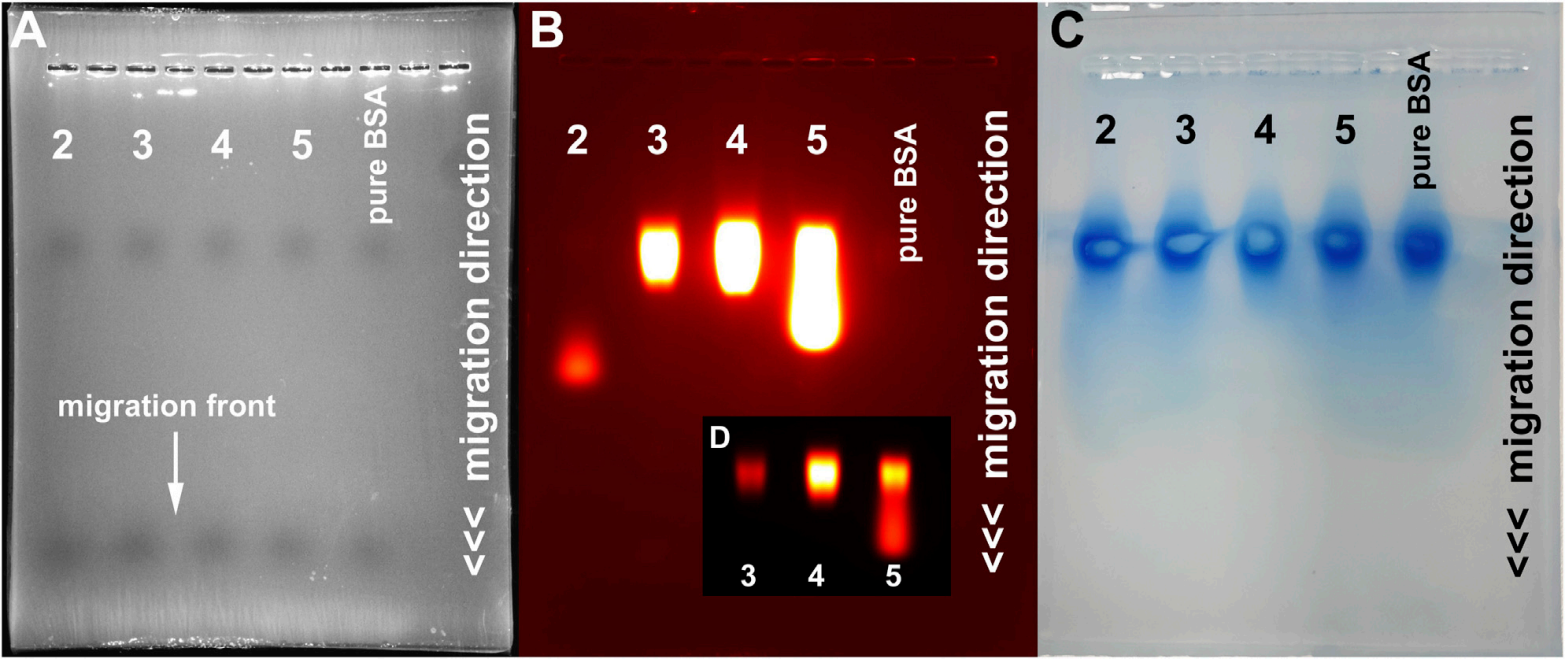
Gel electrophoresis analysis of the four developed probes. **(A)** Phase contrast image showing the migration front (maximum migration distance) of the used lanes (1, 3, 5, 7 and 9). Fluorescence analysis **(B)** shows a markedly reduced migration distance of the modified probes **3**, **4** and **5** (lanes 3,5 and 7) compared to **2**. Coomassie Blue staining **(C)** identifies serum protein located at a reduced migration distance in all lanes. **(D)** Fluorescence image of lanes 3, 5 and 7 at reduced exposure settings, depicting the strongest signal intensity at the BSA level and a certain degree of “spillover” of probe **5**.

First evidence of a high degree of serum albumin binding of the modified probes was apparent after agarose gel electrophoresis of the four probes in the presence of BSA and fluorescence analysis. Figure 2 shows the phase contrast picture (**A**), the fluorescence image (**B**) and the Coomassie Blue stained gel (**C**). Fluorescence analysis shows that the modified probes **3**, **4** and **5** exhibit a marked and equal reduction of migration distance but not the original probe **2**, which migrates about two thirds of the whole distance (compare Figures 2A,B). Protein analysis of the gels by Coomassie Blue staining suggests a co-localization of fluorescence of **3**, **4** and **5** and albumin. The IPBA-modified probe **5** shows a marked degree of “spillover” to a higher migration distance, probably due to reduced binding to BSA. Therefore, we concluded that all modified probes bind to albumin under these conditions, at least to a high degree, while the original probe **2** does not.

**FIGURE 3:**
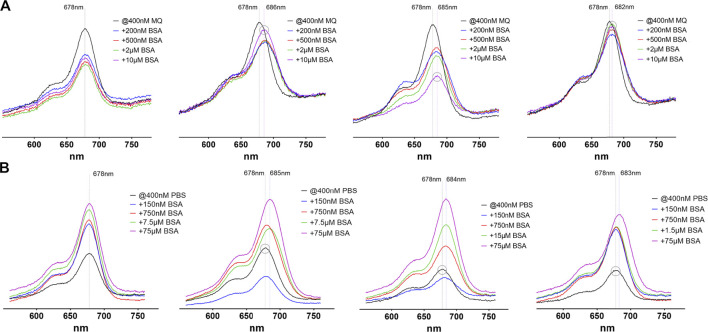
Absorption spectra of the four investigated probes **2**–**5** **(from left to right)** in pure water **(A)** and PBS **(B)**. In each image the black line represents the absorption without addition of BSA, where every compound shows a maximum absorption at 678 nm, regardless of the solvent used. The y-axis scale is identical in all images. The dashed lines depict the absorption maximum wavelengths without and with BSA (maximum red-shift). The grey circle stresses peaks with the highest **(A)** or lowest **(B)** absorption maximum wavelength to illustrate the observed red-shifts.

The spectral characteristics of the probes were examined by photometric and fluorometric measurements in the absence and presence of different bovine serum albumin (BSA) concentrations (Figure 3). We used BSA throughout this study as an archetype serum albumin for its easy accessibility. The absorption spectra of Cy 5.5-labelled probes typically show a maximum at 678 nm and a shoulder at around 630 nm of varying intensity. This shoulder is strengthened by the formation of aggregates, as seen in the absorption spectra of **4**. When analyzing the absorption spectra of compounds **2**–**5** in pure water (Milli-Q®-purified, MQ) it is conspicuous, that with addition of BSA a reduction of the absorption maximum intensity is observed, which in the case of the fatty acid modified compound **4** is markedly concentration dependent and also shows a lowering effect on the intensity of the shoulder, hinting at an initial aggregation. Compound **5** shows the lowest effect, while compound **3** shows a reconstitution of the starting intensity when excess BSA is added. Also striking is a red-shift of the absorption maximum wavelength in the cases of probes **3**, **4** and **5**. This red-shift has been observed before and was attributed to a higher polarity of the dye vicinity, resulting in a lower electron density and therefore a higher stability of the LUMO (lowest unoccupied molecular orbital). This was interpreted as a measure of the degree of serum protein binding and is therefore is not observed for the unmodified probe **2**. When changing the solvent to PBS, the initial absorption is lower for all compounds and shows a concentration-dependent increase when BSA is added. This lower absorption is probably due to a higher degree of aggregation in this solvent. Interestingly, after adding BSA, probes **3** and **4** show a slight decrease in absorption when a substochiometric amount is present, hinting at an even higher degree of aggregation. Increasing amounts of BSA then lead to an overall increase in absorption, roughly to the initial values observed in pure water. Compounds **2** and **5** show an immediate increase in absorption, also with low amounts of BSA. Red-shifts of the absorption maxima of 5–7 nm are also found for compounds **3**–**5** but not for the unmodified probe **2**.

**FIGURE 4:**
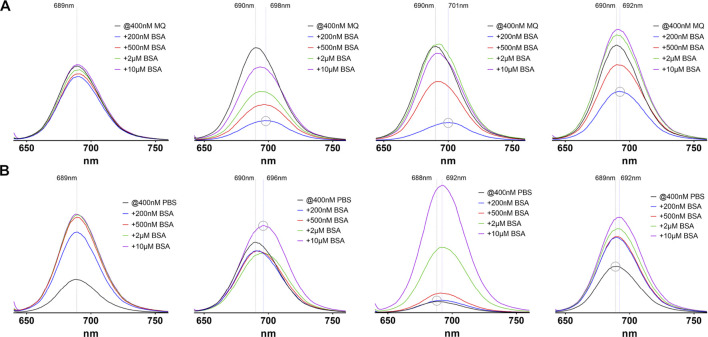
Emission spectra of the four investigated probes **2**–**5** **(from left to right)** in pure water **(A)** and PBS **(B)**. In each image the black line represents the absorption without addition of BSA, where every compound shows a maximum emission around 689 nm, regardless of the solvent used. The y-axis scale is identical in all images. The dashed lines depict the emission maximum wavelengths without and with BSA (maximum red-shift). The grey circle stresses peaks with the highest or lowest emission maximum wavelength to illustrate the observed red-shifts.

Also, the emission spectra of the compounds exhibit striking differences (Figure 4). In MQ water the emission intensity of the albumin-binding compounds **3**–**5** is markedly reduced when low amounts of BSA are present. Raising the amount of BSA up to 25-fold also raises the emission intensity roughly up to the initial values. The albumin binding probes also show a red-shift of the emission maximum of up to 11 nm if BSA is added, resulting from protein binding. This red-shift is maximal when a substochiometric amount of BSA is present (grey circles in Figure 4A) and is then reduced when higher amounts of BSA are added. The unmodified compound **2** does not exhibit this kind of variability. The fact that small amounts of albumin up to a two-fold excess lead to an initial decrease in emission intensity while a larger BSA excess restores the values, may be explained by a first, rather loose attachment of the probes to several different sites and therefore several different energy transfer routes, including quenching between dye molecules. When with higher amounts of albumin only one probe molecule can bind, a single high-affinity site is occupied and energy transfer routes, including vibrational deactivation, are restricted to the local environment. Furthermore, similar to the effect on the absorption spectra, changing the solvent to PBS changes the emission characteristics of all examined probes. The overall emission intensity is reduced compared to measurements in MQ water. This reduction is strongest in **4**, followed by **2**, **5** and **3** and is restored to the values observed in pure water when the amount of BSA reaches a two- to five-fold excess. Compound **4** shows the strongest effect with a more than 10-fold increase in emission intensity.

Overall, the spectrophoto- and fluorometric findings can be interpreted by two main processes. First, there are aggregation processes of the pure compounds. This aggregation is strongly influenced by ionic components in solution, so the PBS-dissolved probes exhibit a clearly noticeable “salt-effect” designated by a reduction of absorption and emission intensities which mainly originates from the cyanine dye since the unmodified probe shows the same effects. Second, aggregates are broken down by binding to serum albumin in solution, especially by tight binding of the ABMs to BSA, as concluded from the observed red-shifts of the absorption and emission maxima, but also compound **2** shows a related, albeit weaker effect on absorption and emission intensities. The aggregation of cyanine dyes in aqueous solution has been intensely studied (v Berlepsch and Böttcher, 2015; Bricks et al., 2017) and also the influence of salt concentrations on spectra of cyanine dyes is well understood (Mooi et al., 2014; v Berlepsch and Böttcher, 2015; Pronkin et al., 2020). However, most examined dyes do not contain many sulfonic acid groups, which in contrast make the molecules investigated in our study highly soluble in aqueous buffers. Therefore, and because of the applied low dye concentrations, we do not suppose a high degree of aggregation of our probes due to cyanine association. The absence of typical dimer or multimer absorption bands like those from H- or J-aggregates as discussed by Bricks et al. contradicts this assumption (Bricks et al., 2017). Rather, the hydrophobic ABMs result in a certain degree of association, especially in high salt concentration. Based on the described spectral characteristics of the examined probes, and considering an *in vivo* application, where a high intravascular concentration of BSA (>600 µM) and salt concentrations comparable to PBS are present, compound **4** stands out a bit, because it is most strongly influenced by changes in BSA or salt levels. This is probably due to the higher number of distinct BSA binding sites for the attached fatty acid and the more physiologic nature of this albumin binding tag.

**FIGURE 5:**
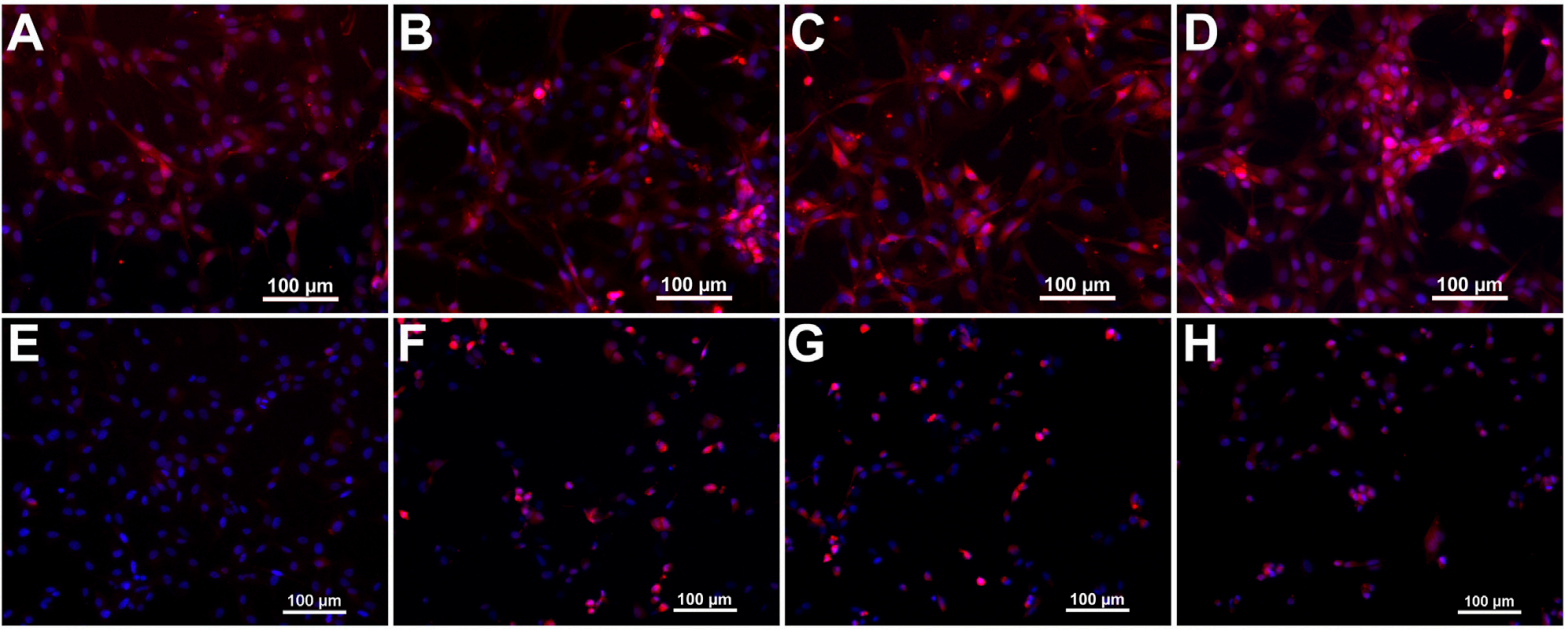
Microscopic images of U87MG cells stained with the developed fluorescent probes. Panels **(A**–**D)** show merged fluorescence images from Cy 5.5 and DAPI channels of probes **2**–**5**, respectively. Panels **(E**–**H)** show cells from control experiments after blocking integrin α_v_β_3_ with an excess of cilengitide.

The developed probes were evaluated concerning their target binding capacity by fluorescence microscopy of integrin α_v_β_3_-expressing U87MG human glioblastoma cells. All probes were able to highlight target expression on cancer cells, showing comparable staining of the cell surface (Figures 5A–D).

Control experiments with an excess of RGD-containing small peptide cilengitide, which was added simultaneous to the probes, yield a marked decrease of cell surface signal intensity. The unmodified probe **2** shows the most evident blocking effect, thus presumably exhibiting the highest specificity (Figure 5E). The ABM-modified probes show a less marked, but still strong reduction in fluorescence signal intensity (Figures 5F–H). Therefore, we presume a conserved integrin target affinity of the designed probes.

**FIGURE 6:**
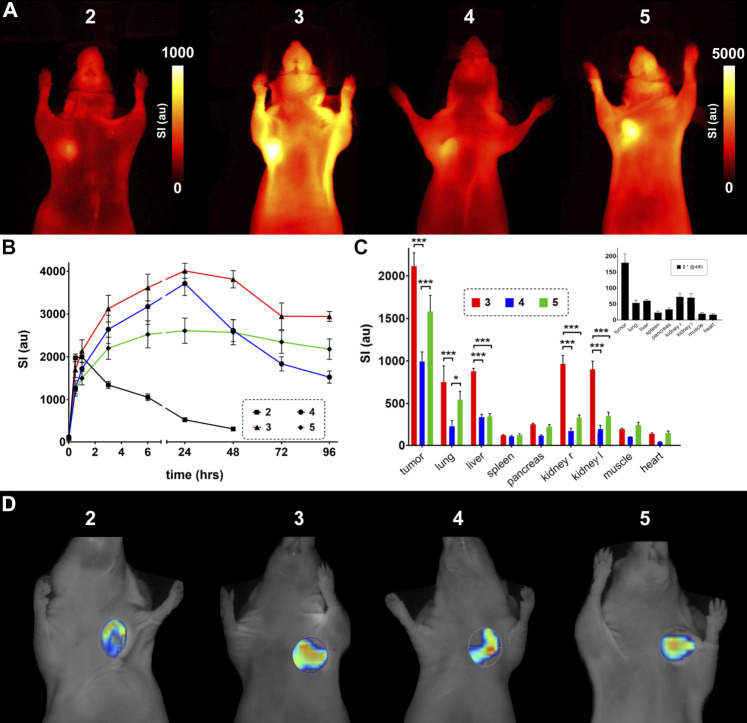
*In vivo* evaluation of the designed probes **2**–**5** in nude mice bearing U87MG xenografts. **(A)** FRI images 48 h after injection of the probe. Note the difference in scale between probe **2** and probes **3**–**5**. **(B)** Timeline of fluorescence signal intensity (au) from tumor tissue *in vivo*. Probe **2** was only investigated for 48 h due to the high signal decay (significant SI differences are listed in Supplementary Table S1). **(C)** Biodistribution of organ fluorescent signal 5 days (96 h) after probe injection (inset, biodistribution of **2** after 48 h). **(D)** Exemplary FMT images including regions of interest around tumor tissue (unscaled).

The *in vivo* investigation of the developed probes included fluorescence reflectance imaging (FRI) and fluorescence mediated tomography (FMT) of α_v_β_3_-expressing U87MG glioblastoma xenografts in nude mice. Subcutaneous xenografts of these tumor cells are well-established models for α_v_β_3_-expressing cancerous lesions (Dumont et al., 2009; Martin et al., 2021). Tumors were imaged for 1 wk after injection of 2 nmol of probe per animal except for the unmodified probe **2**, which could only be imaged for 48 h due to fast washout. The tumor region was accentuated early and showed a clearly enhanced SI throughout the experiment timeline. Figure 6A shows exemplary FRI images 48 h after probe injection highlighting tumor location and signal intensity (SI) differences. The displayed SI of the unmodified probe **2** already shows considerably lower values at this time point. Compound **3** exhibits the highest values followed by **5** and **4**. Extracted data from the different time points is plotted in Figure 6B, emphasizing the fast excretion of **2** and demonstrating the potential of the different ABMs for *in vivo* imaging, which all show a significantly increased tumor SI between three and 48 h post injection. SI of probes **3** and **4** rise significantly higher than SI of **5** after 24 h. Interestingly, probe **4** shows a sharp decrease of SI between 24 and 48 h, indicating an interim conversion or metabolization and resulting in significantly lower SI at 48 h and later-on compared to probe **3**. A summary about the significant SI differences can be found in the supporting information (Supplementary Table S1). Diversities of the applied ABMs are revealed when investigating their influence on organ biodistribution (Figure 6C), which is determined 96 h post injection, except for probe **2**. All three modified probes show a high tumor SI, but especially SI from compound **3** is significantly elevated in liver and kidneys, suggesting still ongoing metabolization and/or excretion processes. Compounds **3** and **5** also exhibit a significantly increased SI in lung tissue compared to probe **4**, which in fact shows significantly lower SI in tumor tissue, but also in excreting organs.

When looking out to potential applications of optical molecular imaging in a clinical setting, like fluorescence guided surgery (Egloff-Juras et al., 2019; Lee et al., 2019), the SI differences between tumor and surrounding tissue is an important determinant. This can be represented by the tumor-to-organ ratios and was analyzed for lung, liver, kidney and muscle tissue after 4 days for probes **3**–**5** and after 48 h for probe **2** (Supplementary Figure S1). Generally, tumor-to-lung ratios are low and do not differ between the four investigated probes. The tumor-to-liver ratios are more variable with higher values for probes **4** and **5** and a very low value for probe **3**, which could already be concluded from the biodistribution results in Figure 6C. The tumor-to-kidney comparison again shows slightly higher values for probes **4** and **5** and lower values for **2** and **3**, while tumor-to-muscle ratios exhibit the highest values for **2**, **3** and **4** and rather low values for compound **5**. So, in this setting probe **4** is the most promising in terms of applicability.

The mechanisms of accumulation within tumor tissue of these fluorescent probes can be divers. First, an enrichment solely due to EPR-mediated albumin accumulation is possible. Second, a mechanism which we propose, by an enhanced target binding due to increased availability within tumor tissue. The EPR effect, however, has recently been discussed very controversially (Nel et al., 2017; Sun et al., 2020). Nel et al. emphasize the incompletely understood biophysical nature of the enhanced permeability in solid tumors and accentuate a dysregulated vascular transcytosis as an important additional factor. Sun and colleagues primarily challenge the translatability of preclinical animal models of EPR-mediated tumor accumulation of nanomedicines to human cancers and underline the need for optimized clinical trials to account for the enhanced efficacy compared to the free drug. Our approach with transiently albumin-binding small molecules does not contain nanoformulations but utilizes endovascular serum albumin for delivery into tumor lesions. The affinity of the RGD mimetic to α_v_β_3_ (Alsibai et al., 2014) is an order of magnitude higher, than the affinity of the ABMs to albumin, which is reported in the micromolar range for related constructs (Zorzi et al., 2019). Integrin target expression within tumor tissue is increased on tumor cells (Desgrosellier and Cheresh, 2010), in tumor-infiltrating immune cells (Dustin, 2019) and on cancer associated fibroblasts (DiPersio and Van De Water, 2019). Therefore, presuming equilibrium conditions and an elevated amount of target α_v_β_3_, the probes would bind the integrin in a reasonable number. The retained specificity and overall performance seen in the *in vitro* investigations (Figure 5) support this mechanism. The lack of accumulation of dye-labelled albumin or albumin transiently labelled with a dye-ABM construct as described in the literature (Hahnenkamp et al., 2014) points towards a rather moderate EPR effect in the chosen tumor model and approves the postulated mechanism of enhanced integrin binding due to higher bioavailability of the modified probes. Another possible explanation for enhanced tumor accumulation of albumin binding probes can be deduced from the observation that serum albumin is utilized by tumors as a nutrient. Albumin binds gp60 on the tumor cell surface and is then internalized and transferred to the lysosome for degradation (Desai et al., 2009). Bound fluorescent probes are liberated and presumably reside within the cells. This would also be the case for covalently bound probes like those described by Usama and colleagues (Usama et al., 2020). In general, the fate of integrin-bound RGD peptides or mimetics after binding their target is not clear, but tumor cells are able to internalize activated integrins (Guo and Giancotti, 2004; Nieberler et al., 2017), therefore paving the way for an accumulation of probes inside target cells.

The applied techniques of small animal fluorescence molecular imaging have been widely used in the monitoring of therapy effects (Vuletic et al., 2017) or enzyme activity (Bremer et al., 2005) and in the investigation of distribution patterns of novel therapeutics, e.g. nanomedicines (Kunjachan et al., 2013). FRI has been shown to be particularly feasible for evaluation of murine xenograft models in nude mice, where absorption and scattering of superficial tissue and fur are negligible (Ntziachristos et al., 2003; Gerwing et al., 2020). FMT can be used for a deeper insight into the murine body (von Wallbrunn et al., 2007; Razansky et al., 2012; An et al., 2018), especially when combined with small animal CT (µCT) in a hybrid system, where a considerable benefit for the accuracy of signal correlation and additional attenuation correction for improved signal quantification can be realized (Rosenhain et al., 2017). Optical imaging of integrin expression has mainly been described in the context of α_v_β_3_-targeted RGD containing peptide probes for cancer-related investigations (Chakravarty et al., 2015; Bernhagen et al., 2019; Zhang et al., 2019; Zhao et al., 2020). The manipulation of biodistribution and bioavailability by utilizing albumin as an endogenous carrier has been exploited in some radionuclide-based imaging approaches (Fischer et al., 2013; Müller et al., 2018).

## Conclusion

The attachment of an albumin-binding moiety to a small molecular RGD mimetic enables fluorescence molecular imaging of integrin expression for a much longer period of time in the chosen tumor model compared to the unmodified fluorescent probe. Significant differences between the investigated albumin binders become obvious when the biodistribution of the developed probes is examined. These include high kidney and liver accumulation of the diphenylcyclohexane-derived probe and a minimal lung accumulation of the fatty acid derived compound. A high SI in the tumor is found for all developed probes, but high SI in excreting organs hamper the overall imaging performance. Thus, a tuning of imaging outcome may be possible by choosing the appropriate ABM. In addition, since the applied modifications enhance the amount of probe that actually reaches tumor tissue, one could envision a future potential of this strategy in therapeutic or theranostic clinical applications.

## Data Availability

The original contributions presented in the study are included in the article/Supplementary Material, further inquiries can be directed to the corresponding author.

## References

[B1] Alsibai W. Hahnenkamp A. Eisenblätter M. Riemann B. Schäfers M. Bremer C. (2014). Fluorescent Non-peptidic RGD Mimetics with High Selectivity for αVβ3vs αIIbβ3Integrin Receptor: Novel Probes for In Vivo Optical Imaging. J. Med. Chem. 57 (23), 9971–9982. 10.1021/jm501197c 25384028

[B2] An Y. Wang K. Tian J. (2018). Recent Methodology Advances in Fluorescence Molecular Tomography. Vis. Comput. Ind. Biomed. Art 1 (1), 1. 10.1186/s42492-018-0001-6 32240398 PMC7098398

[B3] Bernhagen D. Jungbluth V. Quilis N. G. Dostalek J. White P. B. Jalink K. (2019). Bicyclic RGD Peptides with Exquisite Selectivity for the Integrin αvβ3 Receptor Using a “Random Design” Approach. ACS Comb. Sci. 21 (3), 198–206. 10.1021/acscombsci.8b00144 30624885

[B4] Blair H. A. Deeks E. D. (2015). Albumin-Bound Paclitaxel: A Review in Non-small Cell Lung Cancer. Drugs 75 (17), 2017–2024. 10.1007/s40265-015-0484-9 26541764

[B5] Bouchelouche K. Turkbey B. Choyke P. L. (2016). PSMA PET and Radionuclide Therapy in Prostate Cancer. Semin. Nucl. Med. 46 (6), 522–535. 10.1053/j.semnuclmed.2016.07.006 27825432 PMC5123597

[B6] Bremer C. Ntziachristos V. Weitkamp B. Theilmeier G. Heindel W. Weissleder R. (2005). Optical Imaging of Spontaneous Breast Tumors Using Protease Sensing ‘smart’ Optical Probes. Invest. Radiol. 40 (6), 321–327. 10.1097/01.rli.0000163797.23172.90 15905717

[B7] Bremerich J. Bilecen D. Reimer P. (2007). MR Angiography with Blood Pool Contrast Agents. Eur. Radiol. 17 (12), 3017–3024. 10.1007/s00330-007-0712-0 17639407

[B8] Bricks J. L. Slominskii Y. L. Panas I. D. Demchenko A. P. (2017). Fluorescent J-Aggregates of Cyanine Dyes: Basic Research and Applications Review. Methods Appl. Fluoresc. 6 (1), 012001. 10.1088/2050-6120/aa8d0d 28914610

[B9] Chakravarty R. Chakraborty S. Dash A. (2015). Molecular Imaging of Breast Cancer: Role of RGD Peptides. Mrmc 15 (13), 1073–1094. 10.2174/1389557515666150909144606 26349490

[B10] Chamberlain F. E. Jones R. L. Chawla S. P. (2019). Aldoxorubicin in Soft Tissue Sarcomas. Future Oncol. 15 (13), 1429–1435. 10.2217/fon-2018-0922 30873850

[B11] Deberle L. M. Benešová M. Umbricht C. A. Borgna F. Büchler M. Zhernosekov K. (2020). Development of a New Class of PSMA Radioligands Comprising Ibuprofen as an Albumin-Binding Entity. Theranostics 10 (4), 1678–1693. 10.7150/thno.40482 32042329 PMC6993238

[B12] Desai N. Trieu V. Damascelli B. Soon-Shiong P. (2009). SPARC Expression Correlates with Tumor Response to Albumin-Bound Paclitaxel in Head and Neck Cancer Patients. Translational Oncol. 2 (2), 59–64. 10.1593/tlo.09109 PMC2670572 19412420

[B13] Desgrosellier J. S. Cheresh D. A. (2010). Integrins in Cancer: Biological Implications and Therapeutic Opportunities. Nat. Rev. Cancer 10 (1), 9–22. 10.1038/nrc2748 20029421 PMC4383089

[B14] DiPersio C. M. Van De Water L. (2019). Integrin Regulation of CAF Differentiation and Function. Cancers 11 (5), 715. 10.3390/cancers11050715 PMC6563118 31137641

[B15] Dumelin C. E. Trüssel S. Buller F. Trachsel E. Bootz F. Zhang Y. (2008). A Portable Albumin Binder from a DNA-Encoded Chemical Library. Angew. Chem. Int. Ed. 47 (17), 3196–3201. 10.1002/anie.200704936 18366035

[B16] Dumont R. A. Hildebrandt I. Su H. Haubner R. Reischl G. Czernin J. G. (2009). Noninvasive Imaging of αVβ3 Function as a Predictor of the Antimigratory and Antiproliferative Effects of Dasatinib. Cancer Res. 69 (7), 3173–3179. 10.1158/0008-5472.CAN-08-3390 19318569 PMC2749524

[B17] Dustin M. L. (2019). Integrins and Their Role in Immune Cell Adhesion. Cell 177 (3), 499–501. 10.1016/j.cell.2019.03.038 30952447

[B18] Egloff-Juras C. Bezdetnaya L. Dolivet G. Lassalle H.-P. (2019). NIR Fluorescence-Guided Tumor Surgery: New Strategies for the Use of Indocyanine green. Ijn 14, 7823–7838. 10.2147/IJN.S207486 31576126 PMC6768149

[B19] Fischer C. R. Groehn V. Reber J. Schibli R. Ametamey S. M. Müller C. (2013). Improved PET Imaging of Tumors in Mice Using a Novel 18 F-Folate Conjugate with an Albumin-Binding Entity. Mol. Imaging Biol. 15 (6), 649–654. 10.1007/s11307-013-0651-x 23760583

[B20] Garin E. Rolland Y. Edeline J. (2019). 90Y-Loaded Microsphere SIRT of HCC Patients with Portal Vein Thrombosis: High Clinical Impact of 99mTc-MAA SPECT/CT-Based Dosimetry. Semin. Nucl. Med. 49 (3), 218–226. 10.1053/j.semnuclmed.2019.01.006 30954188

[B21] Gerwing M. Kocman V. Stölting M. Helfen A. Masthoff M. Roth J. (2020). Tracking of Tumor Cell-Derived Extracellular Vesicles In Vivo Reveals a Specific Distribution Pattern with Consecutive Biological Effects on Target Sites of Metastasis. Mol. Imaging Biol. 22 (6), 1501–1510. 10.1007/s11307-020-01521-9 32737655 PMC7666295

[B22] Guo W. Giancotti F. G. (2004). Integrin Signalling During Tumour Progression. Nat. Rev. Mol. Cel Biol. 5 (10), 816–826. 10.1038/nrm1490 15459662

[B23] Hahnenkamp A. Alsibai W. Bremer C. Höltke C. (2014). Optimizing the Bioavailability of Small Molecular Optical Imaging Probes by Conjugation to an Albumin Affinity Tag. J. Controlled Release 186, 32–40. 10.1016/j.jconrel.2014.04.053 24815420

[B24] Hamidi H. Ivaska J. (2018). Every Step of the Way: Integrins in Cancer Progression and Metastasis. Nat. Rev. Cancer 18 (9), 533–548. 10.1038/s41568-018-0038-z 30002479 PMC6629548

[B25] Höltke C. Faust A. (2017). Molecular Imaging of Integrins in Oncology. Rmi 10, 17–30. 10.2147/rmi.s96767

[B26] Humphries J. D. Byron A. Humphries M. J. (2006). Integrin Ligands at a Glance. J. Cel Sci 119, 3901–3903. 10.1242/jcs.03098 PMC3380273 16988024

[B27] Hynes R. O. (2002). Integrins. Cell 110 (6), 673–687. 10.1016/s0092-8674(02)00971-6 12297042

[B28] Hynes R. O. Lively J. C. McCarty J. H. Taverna D. Francis S. E. Hodivala-Dilke K. (2002). The Diverse Roles of Integrins and Their Ligands in Angiogenesis. Cold Spring Harbor Symposia Quantitative Biol. 67, 143–154. 10.1101/sqb.2002.67.143 12858535

[B29] Jadvar H. Chen X. Cai W. Mahmood U. (2018). Radiotheranostics in Cancer Diagnosis and Management. Radiology 286 (2), 388–400. 10.1148/radiol.2017170346 29356634 PMC5790308

[B30] Keating G. M. (2012). Insulin Detemir. Drugs 72 (17), 2255–2287. 10.2165/11470200-000000000-00000 23110609

[B31] Kratz F. (2008). Albumin as a Drug Carrier: Design of Prodrugs, Drug Conjugates and Nanoparticles. J. Controlled Release 132 (3), 171–183. 10.1016/j.jconrel.2008.05.010 18582981

[B32] Kunjachan S. Gremse F. Theek B. Koczera P. Pola R. Pechar M. (2013). Noninvasive Optical Imaging of Nanomedicine Biodistribution. ACS Nano 7 (1), 252–262. 10.1021/nn303955n 23067565 PMC3743636

[B33] Leboffe L. di Masi A. Polticelli F. Trezza V. Ascenzi P. (2020). Structural Basis of Drug Recognition by Human Serum Albumin. Cmc 27 (30), 4907–4931. 10.2174/0929867326666190320105316 30894098

[B34] Lee J. Y. K. Cho S. S. Stummer W. Tanyi J. L. Vahrmeijer A. L. Rosenthal E. (2019). Review of Clinical Trials in Intraoperative Molecular Imaging during Cancer Surgery. J. Biomed. Opt. 24 (12), 1–8. 10.1117/1.JBO.24.12.120901 PMC7005471 31808327

[B35] Liu Z. Chen X. (2016). Simple Bioconjugate Chemistry Serves Great Clinical Advances: Albumin as a Versatile Platform for Diagnosis and Precision Therapy. Chem. Soc. Rev. 45 (5), 1432–1456. 10.1039/c5cs00158g 26771036 PMC5227548

[B36] Maeda H. (2015). Toward a Full Understanding of the EPR Effect in Primary and Metastatic Tumors as Well as Issues Related to its Heterogeneity. Adv. Drug Deliv. Rev. 91, 3–6. 10.1016/j.addr.2015.01.002 25579058

[B37] Marelli U. K. Rechenmacher F. Sobahi T. R. A. Mas-Moruno C. Kessler H. (2013). Tumor Targeting via Integrin Ligands. Front. Oncol. 3, 222. 10.3389/fonc.2013.00222 24010121 PMC3757457

[B38] Martin S. Maus S. Stemler T. Rosar F. Khreish F. Holland J. P. (2021). Proof-of-Concept Study of the NOTI Chelating Platform: Preclinical Evaluation of 64Cu-Labeled Mono- and Trimeric c(RGDfK) Conjugates. Mol. Imaging Biol. 23 (1), 95–108. 10.1007/s11307-020-01530-8 32856224 PMC7782405

[B39] Merlot A. M. Kalinowski D. S. Richardson D. R. (2014). Unraveling the Mysteries of Serum Albumin more Than Just a Serum Protein. Front. Physiol. 5, 299. 10.3389/fphys.2014.00299 25161624 PMC4129365

[B40] Mooi S. M. Keller S. N. Heyne B. (2014). Forcing Aggregation of Cyanine Dyes with Salts: A fine Line between Dimers and Higher Ordered Aggregates. Langmuir 30 (32), 9654–9662. 10.1021/la502124b 25073802

[B41] Moreno-Layseca P. Icha J. Hamidi H. Ivaska J. (2019). Integrin Trafficking in Cells and Tissues. Nat. Cel Biol 21 (2), 122–132. 10.1038/s41556-018-0223-z PMC6597357 30602723

[B42] Müller C. Guzik P. Siwowska K. Cohrs S. Schmid R. Schibli R. (2018). Combining Albumin-Binding Properties and Interaction with Pemetrexed to Improve the Tissue Distribution of Radiofolates. Molecules 23 (6), 1465. 10.3390/molecules23061465 PMC6100011 29914162

[B43] Nel A. Ruoslahti E. Meng H. (2017). New Insights into "Permeability" as in the Enhanced Permeability and Retention Effect of Cancer Nanotherapeutics. ACS Nano 11 (10), 9567–9569. 10.1021/acsnano.7b07214 29065443

[B44] Nieberler M. Reuning U. Reichart F. Notni J. Wester H.-J. Schwaiger M. (2017). Exploring the Role of RGD-Recognizing Integrins in Cancer. Cancers 9 (9), 116. 10.3390/cancers9090116 PMC5615331 28869579

[B45] Nowacki T. M. Lenz P. Bettenworth D. Brückner M. Bokemeyer A. Tepasse P. R. (2019). Target-Specific Fluorescence-Mediated Tomography for Non-invasive and Dynamic Assessment of Early Neutrophil Infiltration in Murine Experimental Colitis. Cells 8 (11), 1328. 10.3390/cells8111328 PMC6912230 31661876

[B46] Ntziachristos V. Bremer C. Weissleder R. (2003). Fluorescence Imaging with Near-Infrared Light: New Technological Advances that Enable In Vivo Molecular Imaging. Eur. Radiol. 13 (1), 195–208. 10.1007/s00330-002-1524-x 12541130

[B47] Parmelee D. J. Walovitch R. C. Ouellet H. S. Lauffer R. B. (1997). Preclinical Evaluation of the Pharmacokinetics, Biodistribution, and Elimination of MS-325, a Blood Pool Agent for Magnetic Resonance Imaging. Invest. Radiol. 32 (12), 741–747. 10.1097/00004424-199712000-00004 9406014

[B48] Pronkin P. G. Shvedova L. A. Tatikolov A. S. (2020). Comparative Study of the Interaction of Some Meso-Substituted Anionic Cyanine Dyes with Human Serum Albumin. Biophysical Chem. 261, 106378. 10.1016/j.bpc.2020.106378 32334186

[B49] Rahbar K. Bode A. Weckesser M. Avramovic N. Claesener M. Stegger L. (2016). Radioligand Therapy with 177Lu-PSMA-617 as A Novel Therapeutic Option in Patients with Metastatic Castration Resistant Prostate Cancer. Clin. Nucl. Med. 41 (7), 522–528. 10.1097/RLU.0000000000001240 27088387

[B50] Razansky D. C. Deliolanis N. Vinegoni C. Ntziachristos V. (2012). Deep Tissue Optical and Optoacoustic Molecular Imaging Technologies for Pre-clinical Research and Drug Discovery. Cpb 13 (4), 504–522. 10.2174/138920112799436258 22216767

[B51] Rosenhain S. Al Rawashdeh W. e. Kiessling F. Gremse F. (2017). Sensitivity and Accuracy of Hybrid Fluorescence-Mediated Tomography in Deep Tissue Regions. J. Biophoton. 10 (9), 1208–1216. 10.1002/jbio.201600232 27868394

[B52] Sleep D. (2015). Albumin and its Application in Drug Delivery. Expert Opin. Drug Deliv. 12 (5), 793–812. 10.1517/17425247.2015.993313 25518870

[B53] Su C.-y. Li J.-q. Zhang L.-l. Wang H. Wang F.-h. Tao Y.-w. (2020). The Biological Functions and Clinical Applications of Integrins in Cancers. Front. Pharmacol. 11, 579068. 10.3389/fphar.2020.579068 33041823 PMC7522798

[B54] Sun D. Zhou S. Gao W. (2020). What Went Wrong with Anticancer Nanomedicine Design and How to Make it Right. ACS Nano 14 (10), 12281–12290. 10.1021/acsnano.9b09713 33021091

[B55] Usama S. M. Park G. K. Nomura S. Baek Y. Choi H. S. Burgess K. (2020). Role of Albumin in Accumulation and Persistence of Tumor-Seeking Cyanine Dyes. Bioconjug. Chem. 31 (2), 248–259. 10.1021/acs.bioconjchem.9b00771 31909595 PMC7174984

[B56] v. Berlepsch H. Böttcher C. (2015). H-aggregates of an Indocyanine Cy5 Dye: Transition from Strong to Weak Molecular Coupling. J. Phys. Chem. B 119 (35), 11900–11909. 10.1021/acs.jpcb.5b05576 26244552

[B57] von Wallbrunn A. Höltke C. Zühlsdorf M. Heindel W. Schäfers M. Bremer C. (2007). In Vivo imaging of Integrin ανβ3 Expression Using Fluorescence-Mediated Tomography. Eur. J. Nucl. Med. Mol. Imaging 34 (5), 745–754. 10.1007/s00259-006-0269-1 17131149

[B58] Vuletic I. Zhou K. Li H. Bai H. Meng X. Zhu S. (2017). Validation of Bevacizumab Therapy Effect on Colon Cancer Subtypes by Using Whole Body Imaging in Mice. Mol. Imaging Biol. 19 (6), 847–856. 10.1007/s11307-017-1048-z 28315202

[B59] Weis S. M. Cheresh D. A. (2011). v Integrins in Angiogenesis and Cancer. Cold Spring Harbor Perspect. Med. 1 (1), a006478. 10.1101/cshperspect.a006478 PMC3234453 22229119

[B60] Yardley D. A. (2013). Nab-Paclitaxel Mechanisms of Action and Delivery. J. Controlled Release 170 (3), 365–372. 10.1016/j.jconrel.2013.05.041 23770008

[B61] Zhang L. Shan X. Meng X. Gu T. Guo L. An X. (2019). Novel Integrin αvβ3-Specific Ligand for the Sensitive Diagnosis of Glioblastoma. Mol. Pharmaceutics 16 (9), 3977–3984. 10.1021/acs.molpharmaceut.9b00602 31306580

[B62] Zhao M. Ding J. Mao Q. Zhang Y. Gao Y. Ye S. (2020). A Novel αvβ3 Integrin-Targeted NIR-II Nanoprobe for Multimodal Imaging-Guided Photothermal Therapy of Tumors In Vivo. Nanoscale 12 (13), 6953–6958. 10.1039/c9nr10720g 32191787

[B63] Zorzi A. Linciano S. Angelini A. (2019). Non-covalent Albumin-Binding Ligands for Extending the Circulating Half-Life of Small Biotherapeutics. Med. Chem. Commun. 10 (7), 1068–1081. 10.1039/c9md00018f PMC6644573 31391879

