# Supplementary figures and images for: How Different Albumin-Binders Drive Probe Distribution of Fluorescent RGD Mimetics
Source: Front Chem. 2021 Aug 24;9:689850. doi: 10.3389/fchem.2021.689850 (PMC8421774; doi:10.3389/fchem.2021.689850)

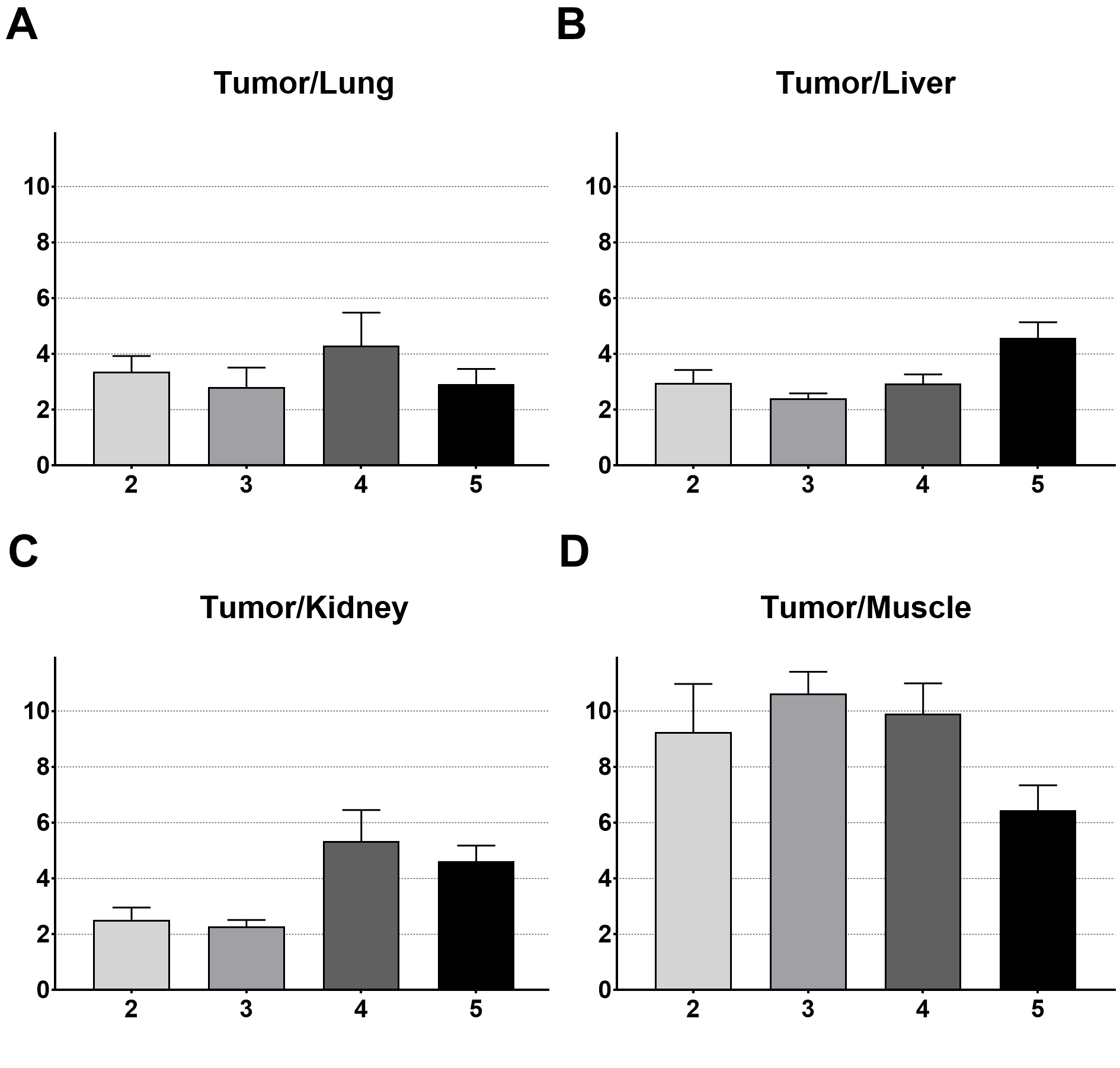

Supplement: Supplementary file 1 [file Image1.JPEG]
