# Supplementary material for: How Different Albumin-Binders Drive Probe Distribution of Fluorescent RGD Mimetics
Source: Front Chem. 2021 Aug 24;9:689850. doi: 10.3389/fchem.2021.689850 (PMC8421774; doi:10.3389/fchem.2021.689850)
Supplement: Supplementary file 2 [file DataSheet1.docx]

**How different Albumin-Binders drive Probe Distribution of fluorescent RGD mimetics**

Carsten Höltke^1*^, Wael Alsibai^1^, Martin Grewer^1^, Miriam Stölting^1^, Christiane Geyer^1^, Michel Eisenblätter^1,2^, Moritz Wildgruber^1,3^, Anne Helfen^1^

^1^ Clinic for Radiology, University Hospital Münster, Münster, Germany;

^2^ Department of Radiology, University Hospital Freiburg, Freiburg, Germany;

^3^ Department of Radiology, University Hospital, LMU Munich, Munich, Germany.

**Supporting Information**

*Syntheses*

All chemicals, reagents and solvents for synthesis were analytical grade and purchased from commercial sources. Fluorescent dye Cy 5.5 was applied as NHS ester and purchased from GE healthcare (Braunschweig, Germany). The synthesis of the unmodified probe **2** as shown in scheme **S1** is described in previous literature ([Alsibai et al., 2014](#_ENREF_1)).

**Scheme S1.** Synthetic route to the unmodified probe **2** showing saponification of **1** followed by Cy 5.5 labelling.

For the synthesis of the diphenylcyclohexyl-modified probe **3**, the methyl ester precursor compound **1** (140 mg, 150µmol), the diphenyl cyclohexyl tyrosine precursor (87.0 mg, 170µmol) as described in the literature ([Hahnenkamp et al., 2014](#_ENREF_3)), TBTU (120 mg, 320µmol) and HOBt (50.0 mg, 320µmol) were dissolved in DMF (5.0mL) and treated with DIPEA (140µL, 800µmol). After shaking overnight, the mixture was diluted with water and the product was extracted with EtOAc. After evaporation the residue was purified by column chromatography (EtOAc/MeOH 4:1). MS analysis (MALDI-TOF/ESI): m/z = 1326.67 [M+H]^+^ (calc.: C_68_H_87_N_9_O_13_SF_3_^+^ = 1326.61). The product was dissolved in 10mL of THF/MeOH/H_2_O (3:1:1) and treated with LiOH (24.0 mg, 1.0mmol) overnight. After removal of the solvent, the residue was directly treated with 10.0mL of 4N HCl in dioxane overnight at rt. The solvent was removed by rotary evaporation and the residue dissolved in water/ACN for HPLC purification. The product-containing fractions were collected and lyophilized (yield: 93.0 mg, 76µmol, 50%). MS analysis (MALDI-TOF/ESI): m/z = 1212.57 [M+H]^+^ (calc.: C_62_H_77_N_9_O_11_SF_3_^+^ = 1212.54). An amount of 8.0 mg (6.6 µmol) of the above precursor was dissolved in 800µL DMF. Then, 5.0 mg of Cy 5.5 NHS ester (4.8 µmol) dissolved in 250µL of DMSO were added, followed by 10µL DIPEA (57µmol). After shaking this mixture for 20h in the dark at rt, the final probe **3** was purified by reversed phase HPLC (H_2_O, acetonitrile, 0.1% TFA, gradient from 70% water to 15% water in 16 min, preparative C18 column, Nucleosil, ø = 20mm, t_R_ = 12 min). MS analysis (Orbitrap/ESI): m/z = 702.5565 [M-3H]^3-^ (calc.: C_103_H_115_N_11_O_24_S_5_F_3_^3-^ = 702.5574), 1054.3364 [M-2H]^2-^ (calc.: C_103_H_116_N_11_O_24_S_5_F_3_^2-^ = 1054.3397).

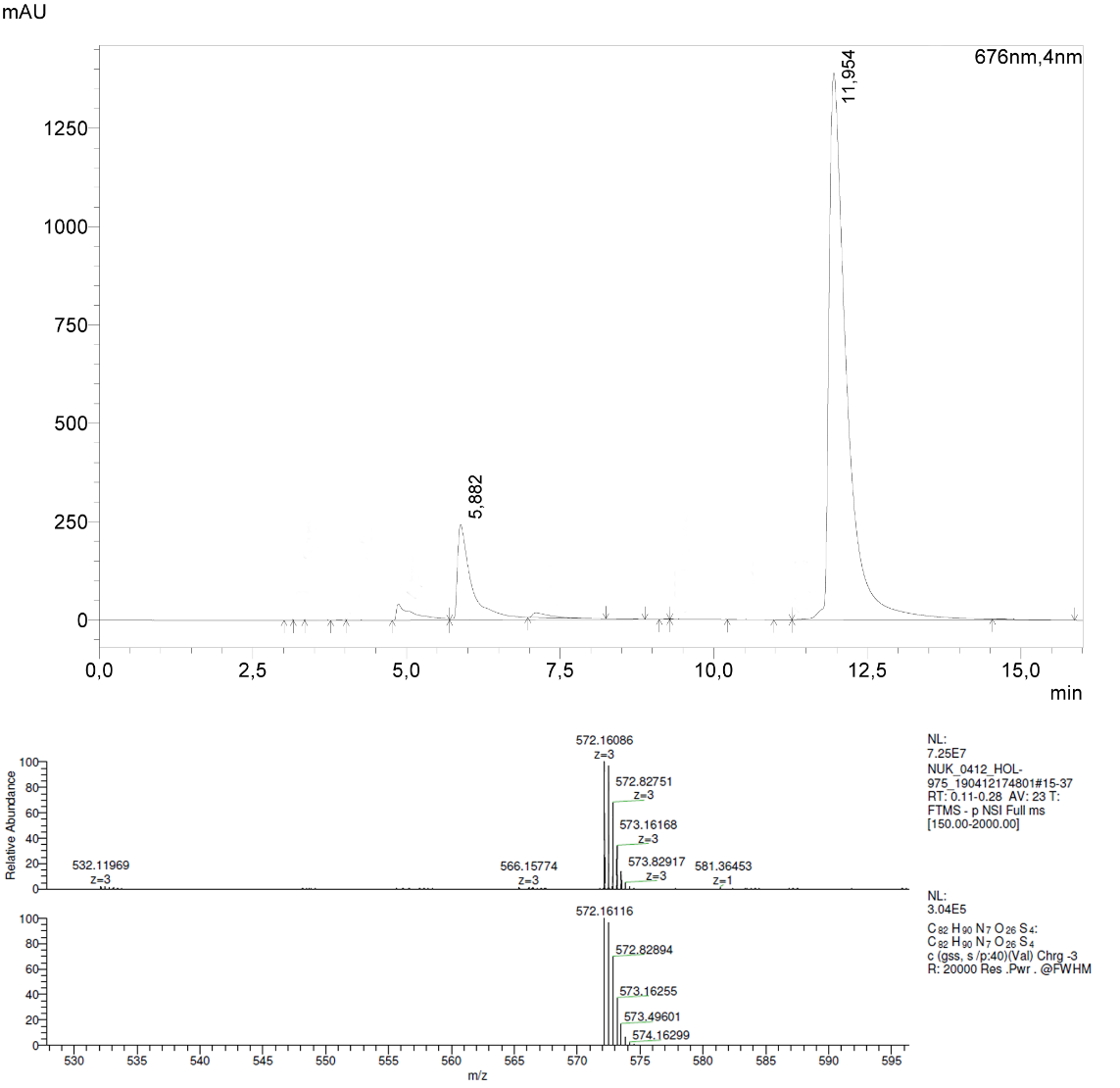


**Scheme S2.** Synthetic route to probe **3**, showing coupling of **1** with the diphenylcyclohexyl-substituted tyrosine derivative, deprotection and labelling with Cy 5.5. The preparative HPLC trace shows the product peak at 12 min and residual free dye at ≈ 6 min. MS analysis mainly detects the triple negative ion of the product.

The Synthesis of the fatty acid derived probe **4** started from the free acid precursor **1'**. An amount of 300mg (620µmol) of Boc-*L*-Lys(Palm)OH (Iris Biotech, Marktredwitz, Germany), 210mg (650µmol) TBTU and 88mg (650µmol) HOBt were dissolved in 10mL of DMF and treated with 175µL (1.0 mmol) DIPEA. After stirring for 30 min at rt, a solution of 500mg (620µmol) of precursor **1'** in 5mL of DMF were added, followed by additional 350µL (2.0 mmol) of DIPEA. Stirring was continued for 17h. Then, water (50mL), EtOAc (20mL) and HOAc (1mL) were added. The product was extracted into the organic phase by additional EtOAc treatment. The organic phase was washed with brine and dried over magnesium sulfate. After removal of the solvent, the residue was taken up in 20mL of 4.0N HCl in dioxane and stirred overnight at rt. The solvent was removed and the residue purified by column chromatography (EtOAc/MeOH 1:1) yielding 420mg (350µmol, 56%) of the desired amino compound. MS analysis (MALDI/ESI): m/z = 1181.66 [M+H]^+^ (calc.: C_57_H_92_N_10_O_11_SF_3_^+^ = 1181.66), 1203.80 [M+Na]^+^ (calc.: C_57_H_91_N_10_O_11_SF_3_Na^+^ = 1203.64). An amount of 5.0 mg (4.2 µmol) of the above precursor was dissolved in 500µL DMF. Then, 2.0 mg of Cy 5.5 NHS ester (2.2 µmol) dissolved in 250µL of DMSO were added, followed by a mixture of 20µL NEt_3_ (150µmol) in 400µL DMF. After shaking this mixture for 20h in the dark at rt, the final probe **4** was purified by reversed phase HPLC (H_2_O, acetonitrile, 0.1% TFA, gradient from 80% water to 30% water in 20 min, semipreparative C18 column, Nucleosil, ø = 8mm, t_R_ = 11 min). MS analysis (Orbitrap/ESI): m/z = 1038.8983 [M-2H]^2-^ (calc.: C_98_H_131_N_12_O_24_S_5_F_3_^2-^ = 1038.9000), 692.2632 [M-3H]^3-^ (calc.: C_98_H_130_N_12_O_24_S_5_F_3_^3-^ = 692.2641).

**
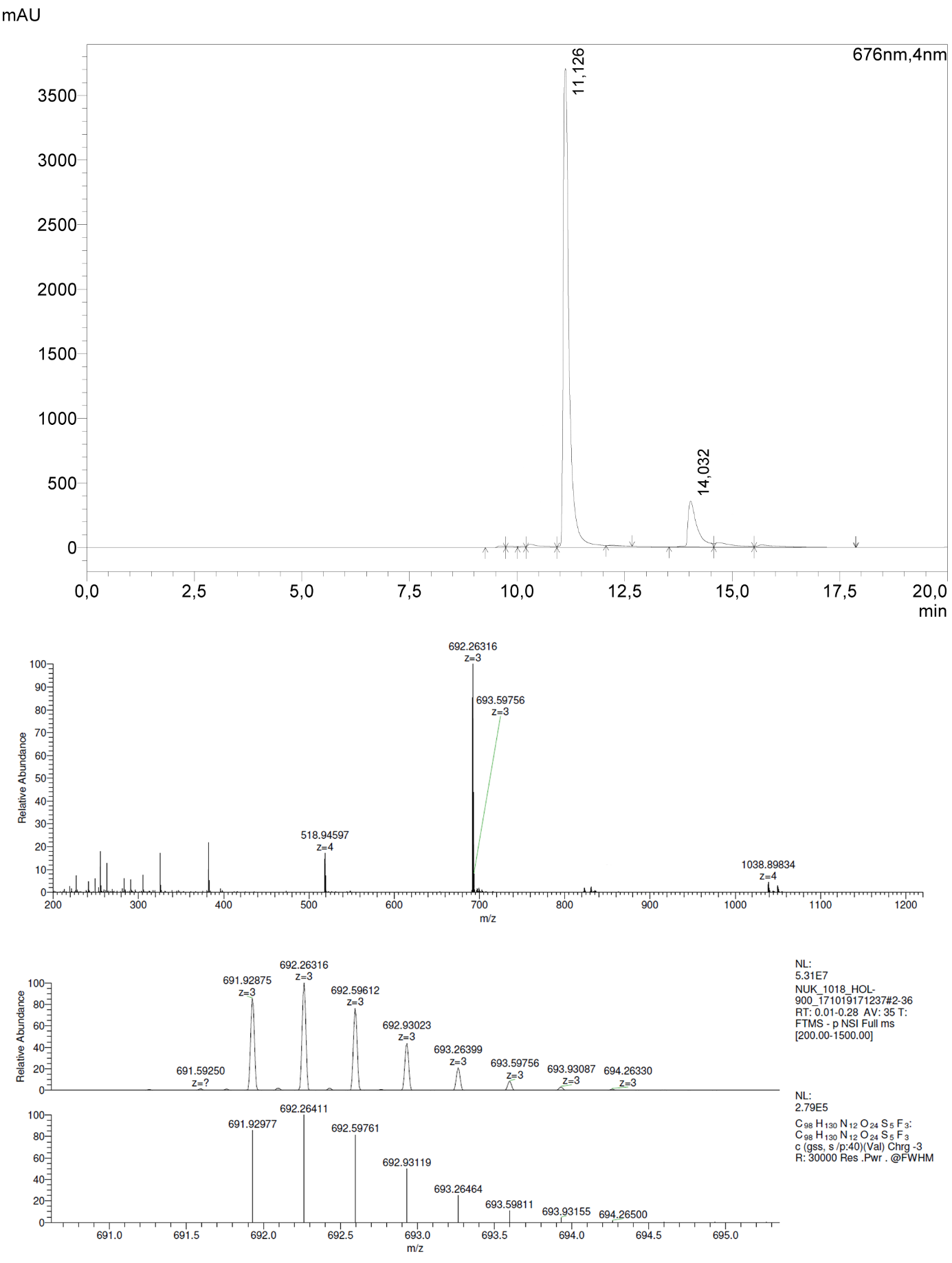
**

**Scheme S3.** Synthetic routes to fluorescent probes **4** starting from the free acid precursor **1'** and Boc-*L*-Lys(Palm)-OH. After removal of the Boc protection the compound was labelled with Cy 5.5 to yield **4**. The preparative HPLC trace shows the product peak at 11 min and unidentified by-product at ≈ 14 min. MS analysis mainly detects the triple negative ion of the product.

For the synthesis of the *p*-iodophenylbutyrate-derived probe **5**, the methyl ester precursor compound **1** (43.0 mg, 50µmol), the *p*-iodophenyl butyric acid lysine precursor (26.0 mg, 50µmol) as described by Dumelin et al. ([Dumelin et al., 2008](#_ENREF_2)), TBTU (32.0 mg, 100µmol) and HOBt (14.0 mg, 100µmol) were dissolved in DMF (3.0mL) and treated with DIPEA (50µL, 280µmol). After shaking overnight, the mixture was diluted with 3mL of pure water and transferred to HPLC purification. The product fraction was evaporated, dissolved in 5mL of THF/MeOH/H_2_O (3:1:1) and treated with LiOH (6.0 mg, 250µmol) overnight. After removal of the solvent, the residue was directly treated with 4.0mL of 4N HCl in dioxane overnight at rt. The solvent was removed by rotary evaporation and the residue dissolved in water/ACN for HPLC purification. The product-containing fractions were collected and lyophilized (yield: 26.0 mg, 22µmol, 44%). MS analysis (Orbitrap/ESI): m/z = 1215.4026 [M+H]^+^ (calc.: C_51_H_71_N_10_O_11_SF_3_I^+^ = 1215.4016), 608.2044 [M+2H]^2+^ (calc.: C_51_H_72_N_10_O_11_SF_3_I^2+^ = 608.2044). An amount of 1.2 mg (1.0 µmol) of the above precursor was dissolved in 500µL DMF. Then, 1.0 mg of Cy 5.5 NHS ester (1.1 µmol) dissolved in 250µL of DMSO were added, followed by a mixture of 20µL NEt_3_ (150µmol) in 400µL DMF. After shaking this mixture for 20h in the dark at rt, the final probe **5** was purified by reversed phase HPLC (H_2_O, acetonitrile, 0.1% TFA, gradient from 80% water to 30% water in 20 min, semipreparative C18 column, Nucleosil, ø = 8mm, t_R_ = 9 min). MS analysis (Orbitrap/ESI): m/z = 1055.77045 [M-2H]^2-^ (calc.: C_92_H_110_N_12_O_24_S_5_F_3_I^2-^ = 1055.76986).

**
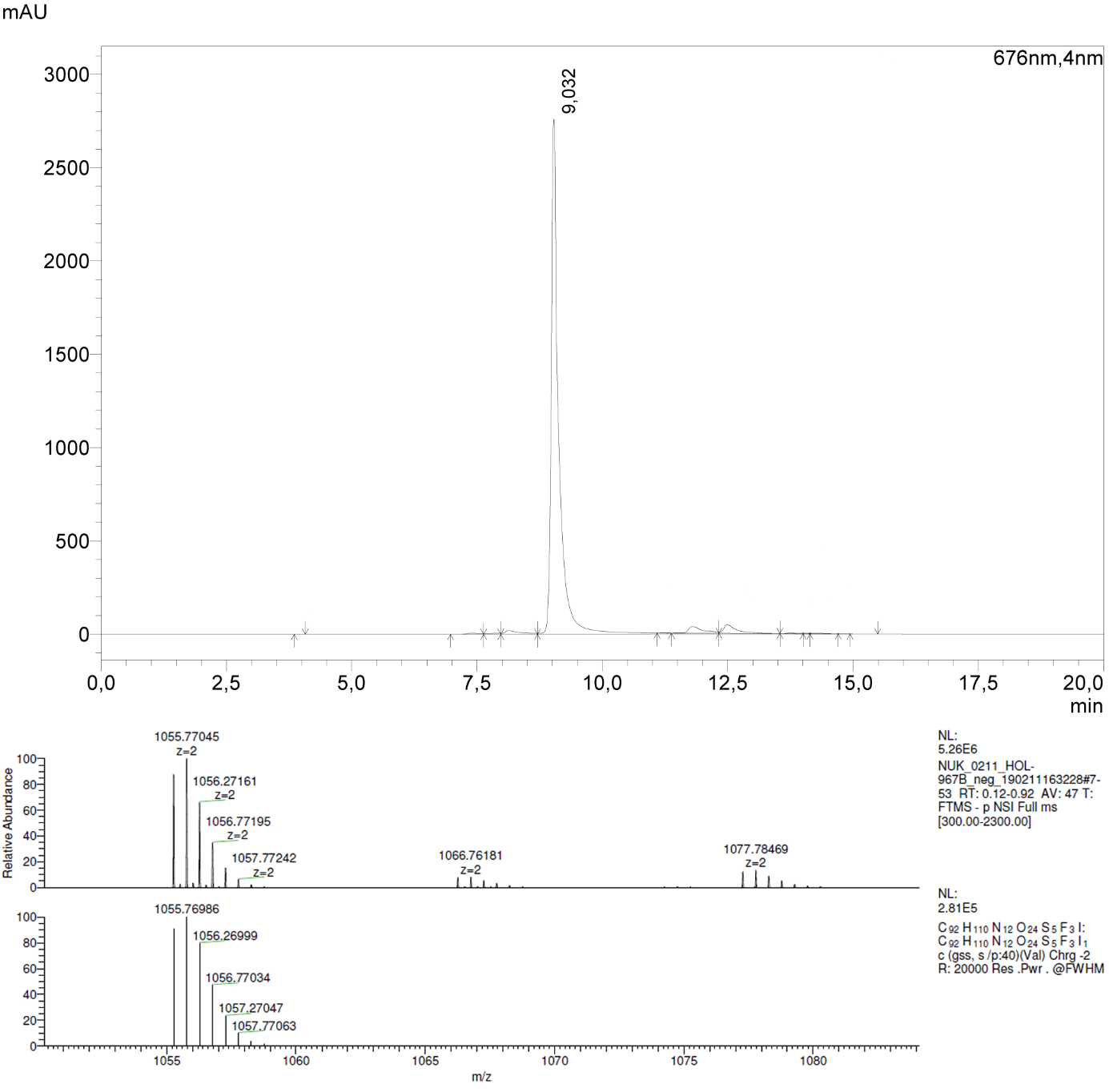
**

**Scheme S4.** Synthetic routes to the developed fluorescent probe **5**. The precursor **1** was coupled with *p*‑iodophenyl butyric acid lysine derivative as described by Dumelin ([Dumelin et al., 2008](#_ENREF_2)). After two deprotection steps the compound was labelled with Cy 5.5 to yield **5**. The preparative HPLC trace shows the product peak at 9 min. MS analysis mainly detects the double negative ion of the product.

*Significant differences of signal intensities from in vivo imaging (p-values)*

|  | **2** vs**. 3** | **2** vs**. 4** | **2** vs**. 5** | **3** vs**. 4** | **3** vs**. 5** | **4** vs**. 5** |
| --- | --- | --- | --- | --- | --- | --- |
| **3 h** | < 0.0001 | < 0.0001 | < 0.01 | ns | < 0.01 | ns |
| **6 h** | < 0.0001 | < 0.0001 | < 0.0001 | ns | < 0.005 | ns |
| **24 h** | < 0.0001 | < 0.0001 | < 0.0001 | ns | < 0.0001 | < 0.005 |
| **48 h** | < 0.0001 | < 0.0001 | < 0.0001 | < 0.005 | < 0.0005 | ns |
| **72 h** | - | - | - | < 0.005 | ns | ns |
| **96 h** | - | - | - | < 0.0005 | ns | ns |

**Table S1.** Table summarizing the p-values of unpaired t-tests performed on the in vivo fluorescence signal intensities of the tumor area at the indicated time-points (FRI measurements, n = 4-8).

*Tumor-to-organ ratios*


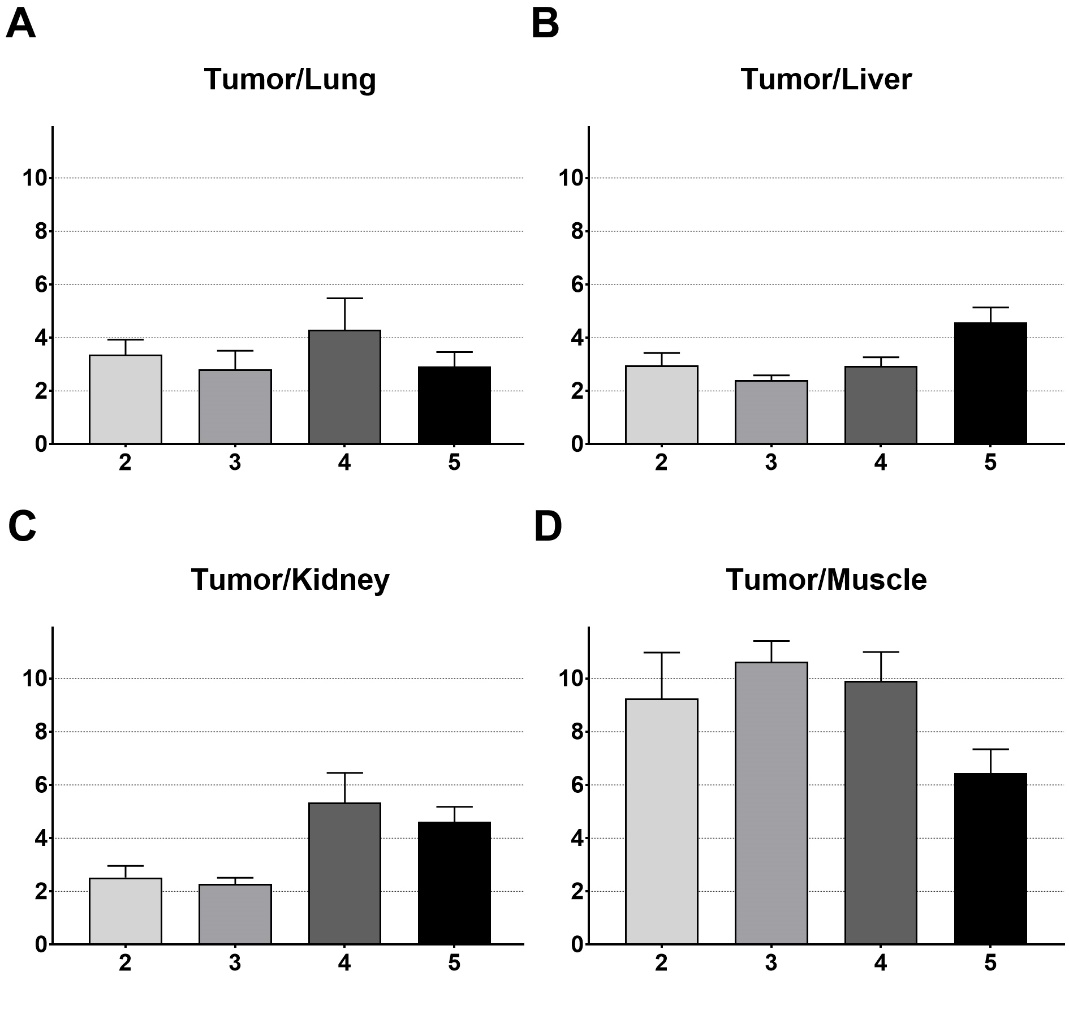


**Figure S1.** Selected tumor-to-organ ratios of signal intensities of the four examined fluorescent probes **2-5** after 48 (probe **2**) and 96 hours (probes **3**-**5**), respectively. Generally, tumor-to-lung ratios are low and do not differ between the four investigated probes. The tumor-to-liver ratios are more variable with higher values for probes **4** and **5** and a very low value for probe **3**. The tumor-to-kidney comparison again shows slightly higher values for probes **4** and **5** and lower values for **2** and **3**, while tumor-to-muscle ratios exhibit the highest values for **2**, **3** and **4** and rather low values for compound **5**.

*References*

Alsibai, W., Hahnenkamp, A., Eisenblätter, M., Riemann, B., Schäfers, M., Bremer, C., et al. (2014). Fluorescent non-peptidic RGD mimetics with high selectivity for alphaVbeta3 vs alphaIIbbeta3 integrin receptor: novel probes for in vivo optical imaging. *J Med Chem* 57(23)**,** 9971-9982. doi: 10.1021/jm501197c.

Dumelin, C.E., Trüssel, S., Buller, F., Trachsel, E., Bootz, F., Zhang, Y., et al. (2008). A portable albumin binder from a DNA-encoded chemical library. *Angew Chem Int Ed Engl* 47(17)**,** 3196-3201. doi: 10.1002/anie.200704936.

Hahnenkamp, A., Alsibai, W., Bremer, C., and Höltke, C. (2014). Optimizing the bioavailability of small molecular optical imaging probes by conjugation to an albumin affinity tag. *J Control Release* 186**,** 32-40. doi: 10.1016/j.jconrel.2014.04.053.
